# Efficacy of Raman Spectroscopy in the Diagnosis of Uterine Cervical Neoplasms: A Meta-Analysis

**DOI:** 10.3389/fmed.2022.828346

**Published:** 2022-05-06

**Authors:** Zhuo-Wei Shen, Li-Jie Zhang, Zhuo-Yi Shen, Zhi-Feng Zhang, Fan Xu, Xiao Zhang, Rui Li, Zhen Xiao

**Affiliations:** ^1^Department of Obstetrics and Gynecology, The First Affiliated Hospital of Dalian Medical University, Dalian, China; ^2^Department of Information Science and Technology, Wenhua University, Wuhan, China; ^3^Department of Gastroenterology, The First Affiliated Hospital of Dalian Medical University, Dalian, China; ^4^The Second Affiliated Hospital of North Sichuan Medical College, Nanchong Central Hospital, Nanchong, China; ^5^Department of Physics, Dalian University of Technology, Dalian, China

**Keywords:** Raman spectroscopy, uterine cervical tumors, diagnostic efficacy, meta-analysis, translational medicine

## Abstract

**Background:**

Uterine cervical neoplasms is widely concerned due to its high incidence rate. Early diagnosis is extremely important for prognosis. The purpose of this article is evaluating the efficacy of Raman spectroscopy in the diagnosis of suspected uterine cervical neoplasms.

**Methods:**

We searched PubMed, Embase, Cochrane Central Register of Controlled Trials (CENTRAL), and Web of science up to September 1, 2021. By analyzing the true positive (TP), false positive (FP), true negative (TN) and false negative (FN) of six included study, we evaluated the pooled and grouping sensitivity, specificity, positive, and negative likelihood ratios (LR), and diagnostic odds ratio (DOR), with 95% confidence intervals (CI), based on random effects models. The overall diagnostic accuracy of Raman spectrum was evaluated by SROC curve analysis and AUC.

**Results:**

After screening with inclusion and exclusion criteria, a total of six study were included in the study. The pooled sensitivity and specificity was 0.98 (95% Cl, 0.93–0.99) and 0.95 (95% Cl, 0.89–0.98). The total PLR and NLR were 21.05 (95% CI, 8.23–53.86) and 0.03 (95% CI, 0.01–0.07), respectively. And the AUC of the SROC curve which show the overall diagnostic accuracy was 0.99 (0.98–1.00).

**Conclusion:**

Through analysis, we confirmed the role of Raman spectroscopy (RS) in the diagnosis of suspected uterine cervical tumors.

**Systematic Review Registration:**

[https://www.crd.york.ac.uk/prospero/], identifier [CRD42021284966].

## Introduction

The incidence rate of uterine cervical tumors is the fourth of female cancer. According to statistics, there were about 570,000 uterine cervical tumors patients and 310,000 deaths worldwide in 2018. Among them, China and India are the hardest hit areas of uterine cervical tumors, accounting for nearly two-thirds of the cases (1). Early diagnosis of cervical cancer and cervical intraepithelial neoplasia and early treatment are effective means to improve the survival rate of cervical cancer patients. Although there are screening tools such as cytological smear (TCT) and human papillomavirus (HPV) detection, the average sensitivity and specificity are not satisfactory (2).

More than 10 years ago, TCT was an effective tool for detecting and preventing uterine cervical tumors. However, the European guidelines for quality assurance of uterine cervical tumors screening (Abstract literature of the Second Edition) released in 2010 pointed out that the false positive rate of cytology is high, which will bring excessive medical treatment and additional economic losses (3). Therefore, HPV DNA detection was recommended due to its high sensitivity. But HPV DNA detection also had the problems of time-consuming and high price. Colposcopy had good sensitivity (>90%), but its specificity was poor (<50%), and the false positive rate was higher, which often lead to unnecessary biopsy. Histopathological examination is the gold standard for the evaluation and diagnosis of cancer, but it includes chemical fixation, dehydration, clearance, infiltration, paraffin embedding, sectioning, and hematoxylin eosin (H&E) staining. It takes about 1 week, which is time-consuming and expensive.

Raman spectroscopy is a new and reliable technology, which can analyze the molecular structure of substances and the chemical composition of human tissues (4). In medical research, Raman imaging has been successfully applied to nasopharyngeal carcinoma (5), gastric cancer (6), lung cancer (7), esophageal cancer (8), renal cell carcinoma (9), brain tumor (10) and so on. Raman technology has been used in the study of uterine cervical tumors for decades. The existing literature has proved that the specificity and accuracy of Raman spectroscopy in the diagnosis of uterine cervical tumors can reach more than 90%, which is no less than the traditional hematoxylin-eosin (HE) staining. Compared with HE staining, Raman technology has the advantages of no staining, no fixation, less demand for professionals, faster and so on, which provides another feasibility for the diagnosis of uterine cervical tumors (11). In conclusion, if Raman spectroscopy can be applied to cervical cancer, we have every reason to believe that it can carry out early diagnosis of cervical cancer and improve the screening rate of cervical cancer and the survival rate of patients. This Meta-analysis reviews the application of Raman spectroscopy in cervical cancer.

## Methods

### Literature Research

This meta-analysis searched PubMed, Embase, Cochrane Central Register of Controlled Trials (CENTRAL), and Web of science to ensure that all potentially eligible articles are included (last search: September 1, 2021). We combined all the relevant medical subject heading (MeSH) terms of uterine cervical tumors and Raman spectrum: [(Uterine Cervical Neoplasms) OR (Cervical Neoplasm, Uterine) OR (Cervical Neoplasms, Uterine) OR (Neoplasm, Uterine Cervical) OR (Neoplasms, Uterine Cervical) OR (Uterine Cervical Neoplasm) OR (Neoplasms, Cervical) OR (Cervical Neoplasms) OR (Cervical Neoplasm) OR (Neoplasm, Cervical) OR (Neoplasms, Cervix) OR (Cervix Neoplasms) OR (Cervix Neoplasm) OR (Neoplasm, Cervix) OR (Cancer of the Uterine Cervix) OR (Cancer of the Cervix) OR (Cervical Cancer) OR (Uterine Cervical Cancer) OR (Cancer, Uterine Cervical) OR (Cancers, Uterine Cervical) OR (Cervical Cancer, Uterine) OR (Cervical Cancers, Uterine) OR (Uterine Cervical Cancers) OR (Cancer of Cervix) OR (Cervix Cancer) OR (Cancer, Cervix) OR (Cancers, Cervix)] AND [(Spectrum Analysis, Raman) OR (Raman Spectrum Analysis) OR (Raman Spectroscopy) OR (Spectroscopy, Raman) OR (Analysis, Raman Spectrum) OR (Raman Optical Activity Spectroscopy) OR (Raman Scattering) OR (Scattering, Raman)]. All potential studies were included with no other limitation. The meta-analysis has been registered in PROSPERO (CRD42021284966).

### Selection Criteria and Exclusion Criteria

Articles like review articles, comments, report, letters will be eliminated from the study. Criteria as follows: (I) without animal tissues in the experiments; (II) reported the use of RS in uterine cervical tumors; (III) used histopathology to confirm the diagnosis; (V) reported the true positive (TP), false positive (FP), true negative (TN) and false negative (FN), based on which the sensitivity and specificity values can be calculated. After screening, a total of six study were included in the study.

### Data Extraction

Two independent investigators extracted a range of data from each study using a standardized data-collecting form: article title, first author, publication year, nationality. All relevant data is contained within the 6 included articles (12–17). Then the primary parameters, which mean the diagnostic value, including TP, FP, TN, and FN. And we can use these parameters to calculate the sensitivity and specificity values. The data obtained were summarized in Table 1.

**TABLE 1:** Characteristics of the included studies.

References	Country		N1	N2	N3	TP	FP	FN	TN	Sensitivity	Specificity	Diagnostic algorithm	Sample	Spectra
Daniel et al. (12)	India	Vitro	25	36	U	23	9	2	27	92%	75%	LDA	Fresh tissue slices (20 μm)	784.12 nm
Daniel et al. (13)	India	Vitro	145	64	U	143	2	2	62	99%	97%	PC-LDA	Fresh tissue slices (20 μm)	784.12 nm
Lyng et al. (14)	Ireland	Vitro	10	20	398	195	2	3	198	98%	99%	PC-LDA	FFPP(10 μm)	514.5 nm
Shaikh et al. (15)	India	Vivo	31	30	154	80	4	0	70	100%	95%	PC-LDA	Cervix *in vivo*	785 nm
Shaikh et al. (16)	India	Vivo	20	6	146	61	3	6	76	91%	96%	PC-LDA	Cervix *in vivo*	785 nm
Jing et al. (17)	China	Vitro	11	11	22	11	1	0	10	100%	91%	ORR (NADH/FAD)	Fresh tissue slices (4 μm)	430 nm

*U, unknown; N1, number of patients; N2, number of healthy; N3, number of tested spectra; FFPP, Formalin-fixed paraffin preserved, PCA, principal component analysis; LDA, linear discriminate analysis; PC-LDA, Principal-component linear discriminant analysis.*

### Statistical Analysis

We calculated the primary data of TP, FP, TN, FN from articles included, then calculated sensitivity, specificity, positive and negative likelihood ratios (LR), based on random effects models. We used Review Man 5.3 and Stata/SE 15.1 to generate the forest plots in order to show sensitivity and specificity.

Meanwhile, Summary Receiver Operator Characteristics (SROC) curves was generated to assess the combination of sensitivity and specificity by Stata/SE 15.1. To assess publication bias, we generated funnel plot using Stata/SE 15.1. In the meantime, we found that articles in uterine cervical tumors include *in vivo* and *in vitro* studies. Therefor we conducted a subgroup analysis according to these studies.

### Risk of Bias (Quality) Assessment

**FIGURE 1:**
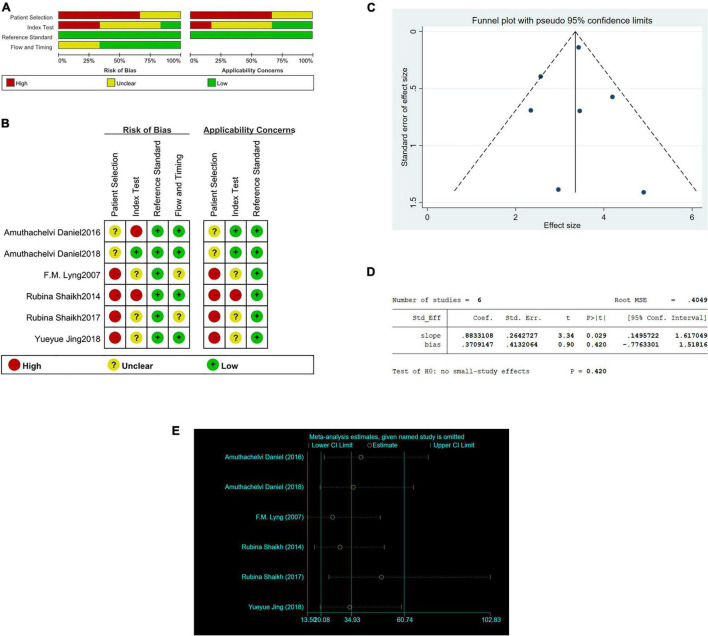
The graphical display of the Quality Assessment of Diagnostic Accuracy Studies (QUADAS-2) of the included studies. **(A)** Risk of bias and applicability concerns evaluation of included studies in pool. **(B)** Risk of bias and applicability concerns evaluation of included studies individually. **(C)** Funnel plot of publication bias in Raman diagnosis of cervical cancer. **(D)** Egger’s regression test of publication bias in Raman diagnosis of cervical cancer. **(E)** Sensitivity analysis in Raman diagnosis of cervical cancer.

Two independent investigators used the Quality Assessment of Diagnostic Accuracy Studies (QUADAS-2) guidelines by Review Manager 5.3 to evaluate the quality of included studies. And the risk of bias of included studies was shown in Figures 1A,B. To assess publication bias, we plotted funnel plots and Egger’s regression test using Stata/SE 15.1. The funnel plots and Egger’s regression test included in the study are shown in Figures 1C,D. As shown in the Figure 1D, *P* = 0.420, less than 0.05, and Egger’s regression test indicates that there is no publication bias.

And we conducted a sensitivity analysis. In Figure 1E, the results showed that none of the studies had an impact on this meta-analysis.

## Results

### Search Results

**FIGURE 2:**
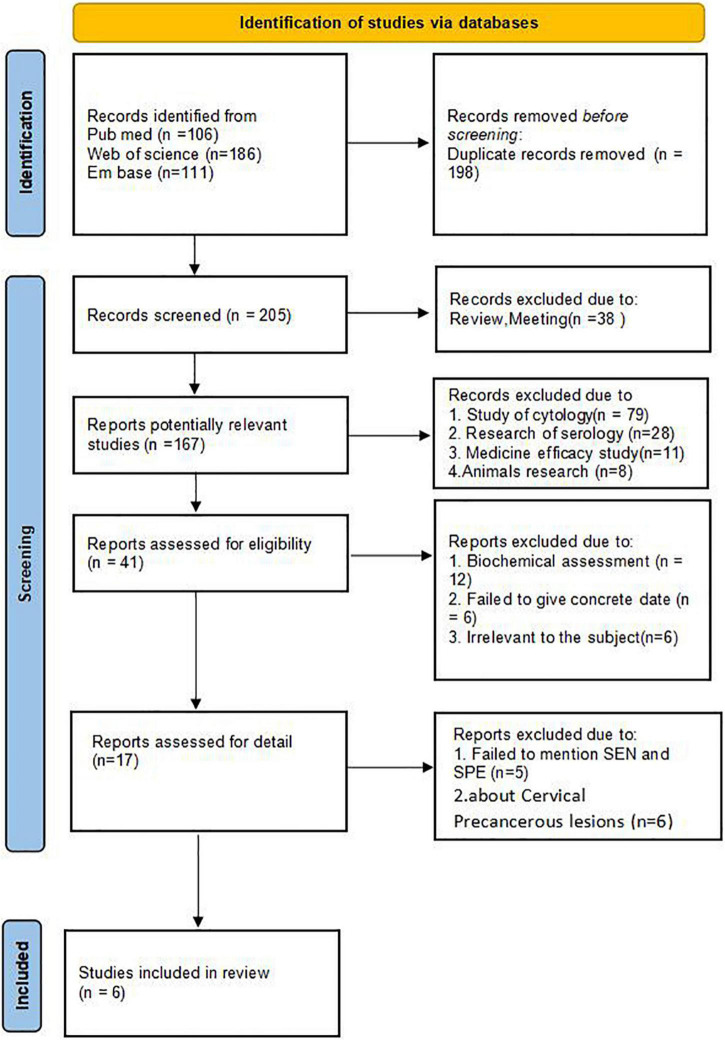
PRISMA 2020 flow diagram.

The process of included articles screening was presented in Figure 2. 403 potential articles were searched at first (including PubMed, *n* = 106, Web of science, *n* = 186, Embase, *n* = 111), in which included 198 duplicate records. Among the rest of 205 articles, 38 articles excluded due to: they were review, meeting or letters. Go a step further by browsing the 167 potentially relevant studies, 126 records excluded due to they were cytological study (*n* = 79), serological research (*n* = 28), medicine efficacy study (*n* = 11), animals research (*n* = 8). By reading the rest of 41 articles, 24 reports excluded due to they were biochemical assessment (*n* = 12), failed to give concrete date (*n* = 6) and irrelevant to the subject (*n* = 6). After careful perusing, 5 articles excluded due to failed to mention TN, FN, TP, FP and 6 excluded because of cervical precancer. Ultimately, 6 studies included in this review.

### Characteristics of the Included Studies

Table 1 carefully described the particular characteristics of the 6 included articles. Among the 6 articles, 5 were published between 2014 and 2018, the rest of article was published in 2007. There are a total of 242 patients and 167 normal people in the included articles, and the total number of spectra incorporated was 720 (two articles didn’t provide the number of spectra). In terms of the nationalities, four studies were from India, other two studies were from China and Ireland, respectively. As for diagnostic algorithm, one article calculated ORR (NADH/FAD), another article used linear discriminate analysis (LDA), and the other four articles utilized Principal-component linear discriminant analysis (PC-LDA). In term of spectra, two studies applied 785 nm, other two studies applied 784.12 nm, and the other two studies applied 430 and 514.5 nm, respectively. All of six studies utilized tissue to research, two studies were *in vivo*, therefor their samples were cervix *in vivo*, and the other four studies were *in vitro*, so their samples were *ex vivo* tissues. Three of four studies *in vitro* obtained fresh tissue slices, the rest of one study obtained Formalin-fixed paraffin preserved tissue.

### Pooled Data Analysis

**FIGURE 3:**
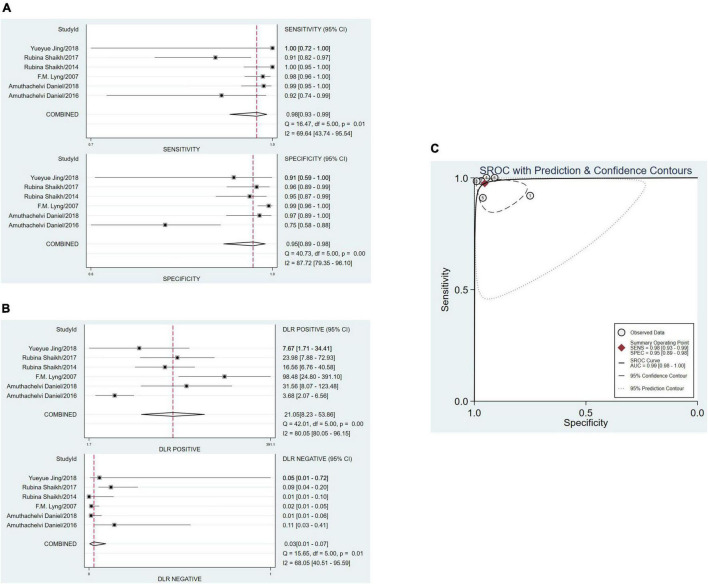
The pooled date analysis of Raman spectroscopy (RS) in uterine cervical tumors. **(A)** The forest plot of pooled sensitivity and specificity of Raman spectroscopy to diagnose uterine cervical tumors of all the six studies. **(B)** The pooled PLR and NLR of Raman spectroscopy in diagnosis of uterine cervical tumors. PLR, positive likelihood ratios; NLR, negative likelihood ratios. **(C)** The SROC curve of Raman spectroscopy in diagnosis of uterine cervical tumors. SROC, summary receiver operator characteristics.

The sensitivity and specificity were calculated to assess diagnostic accuracy of all the six studies. And the forest plot of pooled sensitivity and specificity was shown in Figure 3. The sensitivity which meant the detection of uterine cervical tumors by RS, ranged from 0.91 (95% CI, 0.82–0.97) to 1.00 (95% Cl, 0.95–1.00) and the pooled sensitivity was 0.98 (95% Cl, 0.93–0.99). The sensitivity of all the six studies was more than 0.90, which was mean that the missed diagnosis rate of RS for uterine cervical tumors is very low. The specificity ranged from 0.75 (95% CI, 0.58–0.88) to 0.99 (95% Cl, 0.96–1.00), and the pooled specificity was 0.95 (95% Cl, 0.89–0.98). It should be noted that except for one study with sensitivity of 0.75, specificity of the other five studies were more than 0.90. In a word, the ability of RS to distinguish cancer from normal people was worthy of recognition.

The total PLR and NLR were 21.05 (95% CI, 8.23–53.86) and 0.03 (95% CI, 0.01–0.07), respectively. And the AUC of the SROC curve which show the overall diagnostic accuracy was 0.99 (0.98–1.00). The plots were shown in Figure 3C.

### Subgroup Analysis

#### *Vivo* Group

**FIGURE 4:**
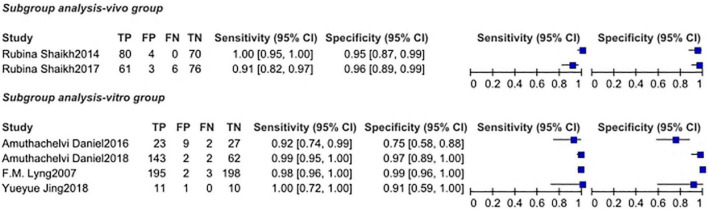
The subgroup analysis of vivo group and *vitro* group.

Two studies (15, 16) showed the research of RS to uterine cervical tumors *in vivo* which had a total of 87 samples and 300 tested spectra. The sensitivity of two studies was 1.00 (95% Cl, 0.95–1.00) and 0.91 (95% Cl, 0.82–0.97), respectively, and the specificity was 0.95 (95% Cl, 0.87–0.99) and 0.96 (95% Cl, 0.89–0.99), respectively. Since the number of study included in this group is less than 4, data analysis cannot be done in STATA. All of the data and grouping situation were shown in Figure 4.

#### *Vitro* Group

**FIGURE 5:**
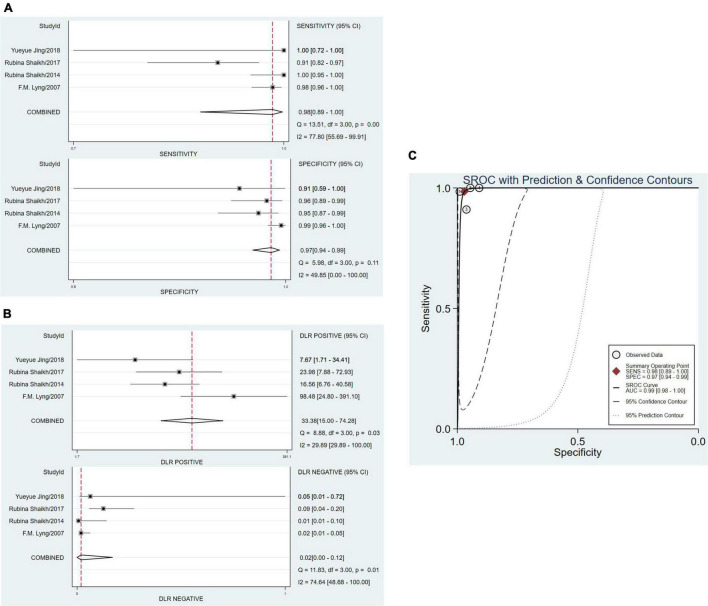
The pooled date analysis of Raman spectroscopy (RS) in uterine cervical tumors *in vitro* group. **(A)** The forest plot of pooled sensitivity and specificity of Raman spectroscopy to diagnose uterine cervical tumors of four studies. **(B)** The pooled PLR and NLR of Raman spectroscopy in diagnosis of uterine cervical tumors. PLR, positive likelihood ratios; NLR, negative likelihood ratios. **(C)** The SROC curve of Raman spectroscopy in diagnosis of uterine cervical tumors. SROC, summary receiver operator characteristics.

Four studies (12–14, 17) showed the research of RS to uterine cervical tumors *in vitro* which had a total of 322 samples and 420 tested spectra (two articles didn’t provide the number of spectra). The sensitivity of four studies ranged from 0.92 (95% Cl, 0.74–0.99) to 1.00 (95% Cl, 0.72–1.00), and the pooled sensitivity was 0.98 (95% Cl, 0.89–1.00). The specificity ranged from 0.75 (95% Cl, 0.58–0.88) to 0.99 (95% Cl, 0.96–1.00), and the pooled specificity was 0.97 (95% Cl, 0.94–0.99). Total PLR and NLR were 33.38 (95% Cl, 15.00–74.28) and 0.02 (95% Cl, 0.00–0.12), respectively. The SROC curve was described and the AUC was 0.99 (0.98–1.00). All of the plots of *vitro* group were shown in Figure 5.

## Discussion

Mahadevan-Jansen et al. first researched uterine cervical tumors *in vivo* and *in vitro* by RS in 1998 (18). That means the research of uterine cervical tumors by RS has had more than 20 years history. Related articles research different substances, such as fresh cervical tissues, cervical cells, blood serum and so on. According to searching, this study is the first meta-analysis attempt to analyze the meaning of RS for uterine cervical tumors by researching fresh cervical tissues, and we intend to confirm its diagnostic accuracy by means of this study.

Meta-analysis showed that RS had high diagnostic accuracy for uterine cervical tumors. The sensitivity of all included articles was more than 90%, and the specificity of most included articles (except for one 75%) were also more than 90%. In the subgroup analysis, the sensitivity and specificity also achieved high standard, that meant whether RS analyze uterine cervical tumors tissues *in vivo* or *in vitro* both showed high diagnostic accuracy. This is strong evidence to explain the diagnostic effect of RS in uterine cervical tumors. Although there are only two literatures *in vivo* subgroup analysis, but for new technologies, such high sensitivity and specificity deserve our attention, and we look forward to seeing more research. And from the perspective of the combination of engineering with medicine, such new technologies and new ideas really deserve our attention.

RS also was used in researching uterine cervical tumors by cervical cells and blood serum except fresh cervical tissue. Sitarz et al. (19) studied the cervical cells of 96 women after TCT and HPV testing. They evaluated Glycogen levels in cells of all study groups to prove that RS can also diagnose HPV infected cells. Karunakaran et al. (20) found that the accuracy of RS in diagnosing uterine cervical tumors and normal people using single cells, cell clusters and DNA were 93.84, 74.26, and 92.21%, respectively. Lu et al. (21) studied the serum of 150 women and detected the levels of SCCA and OPN in the serum by RS. This is a convenient and efficient method which maybe a new screening measure for uterine cervical tumors.

With the prevalence of TCT and HPV examination, pathological biopsy is widely used in clinic and is considered as the gold standard of cervical cancer, what are the outstanding advantages of Raman technology? In other words, how should Raman technology position itself in clinical application?

After reading a lot of literature, people generally believe that the outstanding advantage of Raman microscope lies in its timeliness, such as real-time images, convenience and rapidity, reducing the demand and burden of pathologists and so on. According to the current research progress, Raman technology does not seem to be enough to make us think that it can replace postoperative pathology. However, with the rapid development of modern science and technology, there is an emerging technology called handheld Raman spectrometer, which can quickly and quantitatively detect the anti-cancer drug 5-fluorouracil (5-FU) in serum (22). We have every reason to expect that this technology can be innovated and applied to clinic as soon as possible, such as handheld portable Raman device. This device is smaller, imaging is faster, it is more convenient to determine the scope of lesions, reduce the burden of pathologists, and shorten the time waiting for intraoperative freezing during surgery, so as to realize efficient diagnosis in cost and time.

**FIGURE 6:**
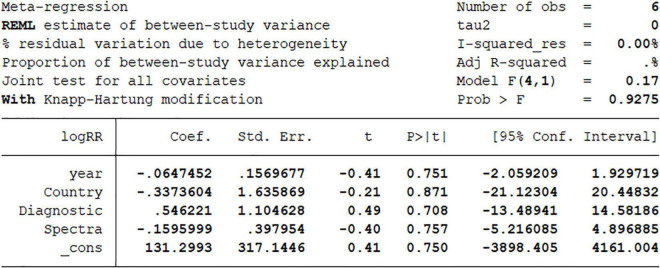
Meta-regression analysis on year, country, diagnostic algorithm, spectra.

There are some limitations in this article. First and foremost, the heterogeneity was high. In order to explore the reasons for this result, we conducted a sensitivity analysis, and the results have been analyzed in Figure 1E. Excluding the included literature one by one did not have a great impact on heterogeneity. And meta regression, grouped by year, country, analysis tool, and Raman wave number, respectively, *P*-values are greater than 0.05, it means no great significance (Figure 6). We believe that the most likely reason is that there is too few research included due to the lack of current research. Second, because the vast majority of studies do not strictly abide by the double-blind test rules when conducting Raman test, there are some errors in the screening of patients, which may affect the analysis results. Third, one of the documents was published in 2007, and the rest were studied in recent 8 years. We don’t know whether microscope technology has developed greatly during this period. However, because there are few articles in conformity, we did not rule it out, and we think this meta can better explain the diagnostic effect of Raman technology in cervical cancer in the past 15 years. If someone continues to choose research in the follow-up, they can directly choose the literature from this time. Fourth, there are only two literatures *in vivo* subgroup analysis. Too few may not directly indicate the effectiveness of Raman technology, which needs more sample size and literature research.

## Conclusion

Due to the high cost and expense of RS, there are not many related studies at present. But in the existing research, it is believed that RS does play an important role in the diagnosis of uterine cervical tumors. This is a satisfactory result which predicts the emergence of a new and efficient diagnostic technology.

Through this meta-analysis, we can confidently believe that Raman spectroscopy has high specificity and sensitivity in the diagnosis of uterine cervical tumors, and we have reason to believe that Raman spectroscopy will become an efficient diagnostic method of uterine cervical tumors in the future. However, more research and evidence are needed to fully demonstrate the role of Raman spectroscopy in the diagnosis of uterine cervical tumors before it is used in clinic. We are also looking forward to more samples and more researches.

## Data Availability Statement

The original contributions presented in the study are included in the article/supplementary material, further inquiries can be directed to the corresponding author/s.

## Author Contributions

Z-WS, L-JZ, Z-YS, XZ, Z-FZ, and FX wrote the manuscript together and made great contributions to the article. This article has taken place under the guidance of two experienced tutors, ZX and RL. All authors agreed to be responsible for the content of this article and the submitted version.

## Conflict of Interest

The authors declare that the research was conducted in the absence of any commercial or financial relationships that could be construed as a potential conflict of interest.

## Publisher’s Note

All claims expressed in this article are solely those of the authors and do not necessarily represent those of their affiliated organizations, or those of the publisher, the editors and the reviewers. Any product that may be evaluated in this article, or claim that may be made by its manufacturer, is not guaranteed or endorsed by the publisher.
